# Captivity and habituation to humans raise curiosity in vervet monkeys

**DOI:** 10.1007/s10071-021-01589-y

**Published:** 2021-12-02

**Authors:** Sofia Ingrid Fredrika Forss, Alba Motes-Rodrigo, Pooja Dongre, Tecla Mohr, Erica van de Waal

**Affiliations:** 1 Department of Ecology and Evolution, University of Lausanne, Lausanne, Switzerland; 2 Inkawu Vervet Project, Mawana Game Reserve, KwaZulu Natal, Pretoria, 3115 South Africa; 3 Department of Evolutionary Biology and Environmental Studies, University of Zurich, Zürich, Switzerland; 4 Department of Early Prehistory and Quaternary Ecology, Eberhard-Karls-Universität Tübingen, Tübingen, Germany

**Keywords:** Curiosity, Novelty response, Neophobia, Exploration, Captivity effect, Captivity bias, Human habituation

## Abstract

The cognitive mechanisms causing intraspecific behavioural differences between wild and captive animals remain poorly understood. Although diminished neophobia, resulting from a safer environment and more “*free*” time, has been proposed to underlie these differences among settings, less is known about how captivity influences exploration tendency. Here, we refer to the combination of reduced neophobia and increased interest in exploring novelty as “*curiosity”*, which we systematically compared across seven groups of captive and wild vervet monkeys (*Chlorocebus pygerythrus*) by exposing them to a test battery of eight novel stimuli. In the wild sample, we included both monkeys habituated to human presence and unhabituated individuals filmed using motion-triggered cameras. Results revealed clear differences in number of approaches to novel stimuli among captive, wild-habituated and wild-unhabituated monkeys. As foraging pressure and predation risks are assumed to be equal for all wild monkeys, our results do not support a relationship between curiosity and safety or free time. Instead, we propose “*the habituation hypothesis*” as an explanation of why well-habituated and captive monkeys both approached and explored novelty more than unhabituated individuals. We conclude that varying levels of human and/or human artefact habituation, rather than the risks present in natural environments, better explain variation in curiosity in our sample of vervet monkeys.

**Supplementary Information:**

The online version contains supplementary material available at 10.1007/s10071-021-01589-y.

## Introduction

Due to both feasibility and logistics, most experimental work on animal cognition is performed in captivity. Nevertheless, cognitive experiments are increasingly being carried out with wild populations in ecologically relevant field settings (Morand-Ferron et al. 2011; van de Waal and Bshary 2011; Thornton and Samson 2012; Benson-Amram et al. 2013; Cauchard et al. 2013; Shaw et al. 2015; Rasolofoniaina et al. 2021). Field experiments usually present wild animals with novel problems in the form of puzzle boxes or devices made of anthropogenic materials. Despite habituation to the apparatuses over time, many studies point to individual differences in neophobia and motivation to participate rather than to differences in cognitive capacities between wild and captive individuals (Overington et al. 2011; Benson-Amram and Holekamp 2012; van Horik et al. 2017; Rössler et al. 2020; Martina et al. 2021). These results suggest that, to successfully implement comparisons of further cognitive skills among settings, we need to improve our understanding of how the motivation to interact and explore novelty differs between captive and wild individuals.

In the broadest sense, curiosity is described as “*the motivation to seek information about something unfamiliar*” (Berlyne 1950; Loewenstein 1994; Byrne 2013; Kidd and Hayden 2015; Gross et al. 2020). This ‘novelty-seeking’ is notably in the absence of any immediate external reward (Wang and Hayden 2019). In humans, psychologists commonly address curiosity through questionnaires and self-reports (see overview in Gross et al. 2020). In non-human animals, however, identifying curiosity requires measures of more specific behavioural components describing readiness and motivation to gather information about something unfamiliar, outside the context of general survival activities (Mettke-Hofmann et al. 2002; Byrne 2013; Hall et al. 2018). Moreover, given the high risks present in most natural environments, many animals have intrinsically strong neophobia, potentially preventing them from engaging in novelty exploration (Barnett 1958; Greenberg 1990a; Mettke-Hofmann et al. 2002). Therefore, it is likely that overcoming neophobia is foundational for when and how wild animals can pursue curiosity driven exploration. Generally, the term neophobia is used to describe “*fear*” of novelty (Greenberg 1990a, b, 2003; Fox and Millam 2007; Greggor et al. 2016a, b), but since we cannot always infer fearful emotions of animals from novel-object test paradigms, the more commonly used definition is “*novelty avoidance*” (Misslin and Cigrang 1986; Benson-Amram et al. 2013; Forss et al. 2015; Greggor et al. 2015; Rasolofoniaina et al. 2021). The contrasting response of closely approaching novel stimuli or preferring novelty over familiarity is termed neophilia (Day et al. 2003; Greenberg 2003; Kaulfuß and Mills 2008). Crucially, one needs to keep in mind that being explorative is *not* the opposite of being neophobic. Instead, explorative behaviours encompass multiple motivational actions relevant to gain information about something unfamiliar (Greenberg 2003; Biondi et al. 2010; Carter et al. 2012; Forss et al. 2017). Therefore, an animal can be both neophobic and simultaneously have a strong exploration tendency (Moretti et al. 2015; Forss et al. 2017). Here, we refer to curiosity as a positive response to novel stimuli expressed through the *combination* of low neophobia (measured as readiness to approach something new) and subsequent explorative behaviours used by an individual to gather knowledge of new encountered stimuli (measured as exploration events, e.g., handling, sniffing, etc.) (Damerius et al. 2017a).

One extreme case leading to reduced neophobia is the risk-free existence of captive animals (Barnett 1958; Brown et al. 2013). The "*captivity effect*" or "*captivity bias*" refers to measurable intra-species cognitive differences between individuals from natural and captive environments (Haslam 2013; Forss et al. 2015; van Schaik et al. 2016; Rössler et al. 2020). Beyond neophobia, a captivity effect has also been described for other behaviours like innovation (Benson-Amram et al. 2013; Rössler et al. 2020) and tool use (Kummer and Goodall 1985; Gruber et al. 2010; Shumaker et al. 2011; Haslam 2013). Variation in activity budgets between wild and captive animals (Veasey et al. 1996; Yamanashi and Hayashi 2011) forms the foundation of the argument that the captivity effect results from wild animals being more occupied with foraging and predator vigilance than captive conspecifics (Kummer and Goodall 1985; Brown et al. 2013; Amici et al. 2020). Accordingly, “*the free time hypothesis”* and “*the excess energy hypothesis”* propose that captive animals have a surplus of energy and a lower cognitive load allowing for higher levels of exploration and innovativeness than wild conspecifics, who are occupied searching for food, mating partners, or shelter (Kummer and Goodall 1985; Laidre 2008a; McCune et al. 2019; Amici et al. 2020). For example, captive hyenas (*Crocuta crocuta*) are less neophobic and more explorative than wild conspecifics, thereby outperforming them in certain problem-solving tasks (Benson-Amram et al. 2013). On the other hand, wild Mexican jays (*Aphelocoma wollweberi*) were faster problem-solvers than captive conspecifics (McCune et al. 2019) and wild-caught and laboratory raised Goffins cockatoos (*Cacatua goffiniana*) differed mainly in their motivation to *participate* in an experimental task, but not in their innovation rates (Rössler et al. 2020). Yet, if and what elements of captive life increase exploration tendencies is less clear. Findings from both primates and birds suggest that frequent exposure to human-made artefacts increases task performance as a result of habituation to artificial materials (Gajdon et al. 2004; Laidre 2008b; van de Waal and Bshary 2011; Damerius et al. 2017a, b). In some primate species, like the great apes, neophobia towards novelty is so high that it can be challenging to perform cognitive tasks through presentation of anthropogenic materials in their natural habitats (Forss et al. 2015; Kalan et al. 2019). Despite being exposed to novel objects for multiple months, wild orangutans (*Pongo abelii* and *Pongo pygmaeus*) only explored them on the rare occasions when they first observed a familiar human interact with the objects (i.e., human presence induced a curious response) (Forss et al. 2015). In captive orangutans, researchers found that individuals' degree of human orientation was positively correlated with exploration tendency, which in turn enhance their problem-solving skills (Damerius et al. 2017b). Thus, it is likely that, in some species, the captivity effect results from human habituation; captive animals show lower neophobia due to reduced risk perception regarding humans, and they develop stronger interest in novelty following increased experience with anthropogenic artefacts (van de Waal and Bshary 2011; Damerius et al. 2017a, b).

In the present study, we examined the foundations of curiosity by investigating neophobia and exploration tendencies in wild and captive vervet monkeys (*Chlorocebus pygerythrus*), using both novel-food and novel-object paradigms. Vervet monkeys are a particularly interesting species to address curiosity as they are opportunistic foragers and successfully inhabit anthropogenic environments like agricultural and urban areas, where they frequently exploit human food sources (Wimberger and Downs 2010; Thatcher et al. 2019). As a highly generalist and “nuisance” species, we expect them to show low levels of neophobia and high exploratory tendencies towards novel stimuli (Greenberg 2003; Sol et al. 2011; Tryjanowski et al. 2016; Griffin et al. 2017; Barrett et al 2019; Jarjour et al. 2020).

Specifically, we aimed to investigate whether curiosity in vervet monkeys is related to habituation to humans or due to low environmental risk and increased free time per se. In the first case, we compared the responses to novel stimuli of captive monkeys to those of wild habituated and wild unhabituated individuals. We predicted that if there existed a captivity effect, wild monkeys (habituated and unhabituated) would show less interest in unfamiliar objects and foods than captive conspecifics. To address the influence of human habituation on curiosity, we performed a separate test to compute the habituation index of each habituated vervet group. We predicted that groups with higher habituation indices would show more curious responses towards the battery of novel stimuli. In addition, for the wild-habituated monkeys, we evaluated whether the habitat structure of the location where the experiments were conducted had any influence on the monkeys’ responses. Here, our prediction was that certain habitat structures, like high grass or open savannah, possibly impose higher predation risk and that monkeys would therefore be less motivated to explore in these habitat structures, compared to when the experiments were performed underneath a tree, providing a more protected location. Because sociality is expected to reduce risk perception and the presence of group members has been shown to increase approaches to novel objects in other species (Stöwe et al. 2006; Moretti et al. 2015; Forss et al. 2017), we predicted that in riskier habitat structures, monkeys would approach more in a social context, accompanied by one or more group members. Finally, given that captive and wild monkeys vary in their experiences with human-made artefacts, we used foods and objects of natural and artificial characteristics to evaluate any potential effect of stimuli features.

## Methods

### Subjects and study sites

We collected data on wild vervet monkeys (*Chlorocebus pygerythrus*) during February and March 2020 at the Inkawu Vervet Project (IVP) field site, located in Mawana game reserve (28° 00.327 S, 031° 12.348 E) in KwaZulu-Natal, South Africa. The study site is home to multiple wild groups of vervet monkeys, six of which are habituated to humans, regularly observed by researchers, and partake in experimental studies. Our data set comprised four of these groups, three of which are habituated since 2010 (Baie Dankie: *N* = 57, Noha: *N* = 39, Lemon Tree: *N* = 24) and the fourth since 2013 (Kubu: *N* = 19). In addition, the study area sustains at least three unhabituated groups, with many more living throughout the rest of the reserve. To enable data collection on unhabituated monkeys and to record any potential interactions with the novel stimuli, we placed motion-triggered video camera traps below two known sleeping trees of an unhabituated group (Congo: *N* = 11).

We collected data on the captive population in March 2020 at the Wild Animal Trauma Centre & Haven (WATCH) vervet sanctuary, in Vryheid, KwaZulu Natal, South Africa. At the time of data collection, the WATCH sanctuary housed three groups of vervet monkeys. For logistical reasons, we only included two groups in our study (Poena: *N* = 17 and Boeta: *N* = 3). Most of the monkeys arrived at the sanctuary and were cared for by humans, since they were a few weeks old, and only a few individuals arrived at a later life stage. At first, infant monkeys arriving at a very young age are housed indoors and bottle nursed by human caretakers. Once they reach 3 months of age, they are slowly integrated into a group of conspecifics of mixed ages. Since the goal is to release these individuals back into their natural habitat (if circumstances allow), caretakers, and occasionally researchers, limit their contact with the monkey groups as much as possible.

### Experimental setup

#### Habituated groups

We presented all four habituated groups with eight novel stimuli representing distinct materials, structures, and odours. We categorized four of these items as human-made or processed: boiled pasta (green, red, natural coloured), popcorn, toy mice (with Baldrian herb scent), and plastic toy cars (yellow, blue, green, and red). One item, white seashells, represented a completely natural occurring object. We chose the remaining three items to have “naturalistic features”: fish (dead organic material in form of canned sardines), beef meatballs (raw organic material), and rubber butterflies of different colours (man-made material which mimics naturally occurring organisms) [Supplementary information (SI) Fig.S1]. We randomized the order of presentation of the novel stimuli across groups to avoid order effects and presented one type of novel stimulus at a time, on the ground, always with several items of each type to avoid potential monopolization by higher ranking group members. To attract the wild monkeys’ attention to the experimental area, prior to the start of the experiment, we placed a handful of familiar food (corn) in the middle of the area where the novel items were spaced out. The habituated monkeys are used to eating corn as this food item has been introduced during both the habituation process as well as during previous experimental studies (van de Waal et al. 2013; van de Waal et al. 2017). Our main goal was to record any potential behavioural reactions towards the novel stimuli after the monkeys had been attracted to the area (within 20 m) and thus seen the novel stimuli. We did all experiments during the early mornings 1–2 h after dawn and we presented only one category of novel stimuli per group per day. We video recorded all experiments with Sony handycams HDR-CX200, two mounted on tripods from different angles, and a third that was handheld by an observer zooming in on any observed explorative behaviours. We presented all novel stimuli to the monkeys for 20 min, to allow enough time for lower ranking individuals to also approach in case the most dominant individuals were present at the start of the experiment preventing the lower rankers from approaching. Because the microhabitats vary slightly across groups as well as within each groups’ home range, depending on their location on the day of our experiments, we categorized each experimental setup into three distinct habitat structures: open savannah (no canopy protection and no high grass), high grass (high grass but no canopy protection), and below tree (the experimental area was protected by canopy). In the open savannah, vervet monkeys are exposed to aerial predators like eagles and monkeys are observed to restrict their movement in high grass as the study area is home to a high abundance of pythons, capable of capturing vervet monkeys. Consequently, below trees represents the safest habitat structure for the monkeys as the tree canopy serves as protection from aerial predators and these areas do not have high grass.

#### Unhabituated group

The unhabituated group would not tolerate any human presence, as individuals from this group run away when human observers approach. They were however already habituated to eat corn when placed out in their habitat. We used an identical set up as with the habituated group, where we placed a small amount of corn in the middle of the area with the novel items. To record data from the unhabituated group, we placed the video camera traps in a way that they captured two different angles of the novel items, which we presented to the monkeys below two of their known sleeping trees. We used all the same novel stimuli as those used for the habituated groups. Because of the uncertainty regarding when the group would pass by the experimental location or when the monkeys would exactly use those sleeping trees, we presented the novel stimuli for 2 days in a row (unless a recording of any approaches by the group took place before the end of 2 consecutive days). Recordings from the camera traps thus allowed us to distinguish whether the group approached the novel stimuli on a single or multiple visits. For comparisons with the other group types, we only used the responses observed during the first visit.

#### Captive groups

At the WATCH sanctuary, we placed the novel stimuli in the main enclosure of the monkeys, who we moved into a side enclosure during the preparation of the experiment, and then let back into their normal enclosure. We used the same experimental protocol as for the habituated groups, including categories and numbers of novel stimuli, experimental duration, video camera placements, and recordings. As the captive monkeys were not used to corn, we used a few peanuts instead as the familiar food that would attract their attention to the experimental area.

### Video coding and measurements

We coded all behavioural responses from video recordings. We recorded the number of close proximity approaches—those made to within 1 m of any of the multiple novel stimuli (food or item)—by any monkey that was present within a 20-m radius of the experimental location. As we defined a close proximity approach as each time a monkey approached within 1 m the novel stimuli, in any case where a monkey left the experimental area and then approached within 1 m again, this represented two approaches. For each approach that was made to the experimental area, we also distinguished whether or not the approach was made alone (when no other monkey was present within 1 m of the novel stimuli) or socially (when there was at least one other monkey present within 1 m of the novel stimuli). Once a monkey made physical contact with a novel item (0 m), we coded following exploration events: the number of smelling and tasting events, the number of times a monkey touched the novel item by hand, the number of times when a monkey chewed/bit the novel stimuli and the number of times a monkey lifted and moved an item. We then summed these behaviours into one exploration score labelled *number of exploration events* for each group and item. For each novel-food item, we additionally scored whether or not a monkey tasted it, defined as an event where a monkey licked a novel-food item, or every time a monkey put its lips onto a food item without ingesting it. All definitions of the coded behaviours as well as the frequencies of approaches and exploration events per group can be found in the ethogram in Table S1 and Fig. 4S in the Supplementary material.

### Habituation test

To estimate the variation in human habituation among groups, we additionally performed a habituation test with the wild-habituated and captive groups. During this test, we exposed the monkeys to a human male that they had never seen before. The wild-habituated groups are familiar with researchers and project volunteers who attempt to distinguish themselves from other humans such as poachers by always wearing a turquoise blue cap while in the presence of the monkeys. In the habituation test, the man was dressed all in black clothes and wore a black cap. The man walked calmly towards the group of the monkeys shaking a Tupperware with corn as this is a familiar signal to the monkeys when they participate in research experiments. In the wild setting the man then placed the closed box with corn at his feet and as a group level habituation index, we measured the proportion of monkeys that approached the man to a distance of 1 m out of all the monkeys present within 20 m. In the captive setting, the man placed himself right at the enclosure mesh and placed peanuts right at his feet, which were in touchable distance to the monkeys. This test lasted 20 min in total.

### Statistical analyses

We conducted the statistical analyses in R (version 3.6.1; R Core Team, 2020) and RStudio (version 1.2.5031; RStudio Team, 2020). We z-transformed covariates (habituation index and group size) to have a mean of zero and standard deviation of one before including them in the models to facilitate the interpretation of the coefficient estimates (Schielzeth 2010).

We first conducted a series of Spearman correlations to investigate whether any of the response measures (number of close proximity approaches, number of exploration events, and number of individuals within each group that tasted the food items) were correlated (Table 2). Since the number of individuals that tasted the different stimuli strongly correlated with the other response measures and this variable contained multiple missing values (*N* = 24), we excluded this variable from further analysis.

To address the study aims, we fitted four different Generalized Linear Mixed Models (glmm) to the data (Table 1). We checked all models (Model 1a,1b, 2, 3) for overdispersion and overall stability (see Supplementary material) and z-transformed continuous variables (Habituation index and group size) before including them as fixed effects (Table 1). We draw inference by comparing the full model with a reduced (null) model lacking the predictors of interest but containing all other model elements (Forstmeier and Schielzeth 2011) using a likelihood ratio test (test “Chisq”' in the R function anova, (Dobson 2002). We implemented this approach to avoid “cryptic multiple testing” and to maintain type 1 error rates at the desired nominal level of 0.05 (Forstmeier and Schielzeth 2011). We calculated individual p values for each predictor using the function drop1 and R squared using the function r.squaredGLMM.

**Table 1:** Descriptions of the different model structures. Variables preceded by a “*z*” indicate that this variables were z-transformed before being introduced in the models

Model	Response variable	Fixed effects	Random effect	Offset
1a	Number of approaches	Stimuli type (8 levels); Group type (3 levels)	Group ID (7 levels)	Log group size
1	Number of approaches	Stimuli type (8 levels); Group type (2 levels)^a^; z-Habituation index	Group ID (6 levels)	Log group size
2	Number of exploratory events	Stimuli type (8 levels); Group type (2 levels)^a^; z-Habituation index	Group ID (6 levels)	Log group size
3	Two-column matrix including number of social approaches and number of individual approaches per trial	Stimuli type (8 levels); Habitat structure (3 levels); z-Habituation index; z-Group size^b^	Group ID (4 levels)^2^	–

Group size was log-transformed before being introduced as an offset

^a^Wild habituated groups were excluded from the model as they did not pose a habituation index

^b^Included as control predictor

In the first model (Model 1a), we investigated the effects of group type (three levels: wild habituated, wild unhabituated, and captive) and stimuli type (8 levels, see above) on the number of approaches (response variable, count data) observed in a given group. For Model 1a, which had a Poisson error structure and log-link function, we fitted the function glmer from the package lme4 (Bates et al. 2014). To account for group identity, we included the random intercept of group ID (7 levels, see above) into the model. We also included the logarithm of group size as an offset term to account for the different number of individuals in each of the groups.

To evaluate variation in close proximity approaches in relation to habituation level, we fitted a second model (Model 1b) using the same response variable, random structure, and the same offset as in Model 1a, but we changed the fixed effect structure. In addition to group type (2 levels: wild habituated and captive) and object type, we included the habituation index into Model 1b. As unhabituated groups did not have a habituation index, we excluded this group from those models where this variable was included (Model 1b and Model 2, see below).

In Model 2, we evaluated potential differences in explorative behaviour among the different group types (2 levels: wild habituated and captive), habituation level and stimuli type (8 levels). Similar to Models 1a and 1b, in Model 2, we included the random intercept of group ID as well as group size as an offset. To avoid overdispersion problems, we fitted this model using a quasi-Poisson model with a negative binomial distribution and the optimizer “bobyqa”.

In Model 3, we evaluated whether the proportion of social approaches varied according to habitat structure, habituation level, and stimuli type. In Model 3, we only included data from wild-habituated groups as these groups were the only ones that had been tested at locations with different habitat structures (below tree: *N* = 14, high grass: *N* = 10, and open savannah; *N* = 13). Model 3 was a binomial model with a response variable in the form of a matrix containing two columns corresponding to the number of social approaches and the number of individual approaches per trial (Baayen et al. 2008). Using such response variable, we account for the different number of approaches observed in different trials. Given that binomial models do not allow including offsets, we included group size as a control predictor. As before, we also included the random intercept of group ID was included in the model (although note that in this case group ID only had 4 levels, which is the threshold generally used to substitute a fixed by a random effect, meaning that it could have also been included as a control predictor).

## Results

### Relationship between response measurements

We found that all three response measures were significantly correlated among one another. The strongest correlation was found between the number of individuals tasting the novel stimuli and the number of exploratory events observed in a group. Correlation coefficients and *p* values of the correlations can be found in Table 2.

**Table 2:** Coefficients and p values in parenthesis resulting from the correlation analyses performed among curiosity measures

	*N* approaches	*N* exploratory events	*N* tasting individuals
N approaches	1	0.47 (< 0.001)	0.45 (0.01)
N exploratory events	–	1	0.61 (< 0.001)
N tasting individuals	–	–	1

### Factors influencing approaches to novel stimuli

**Fig. 1:**
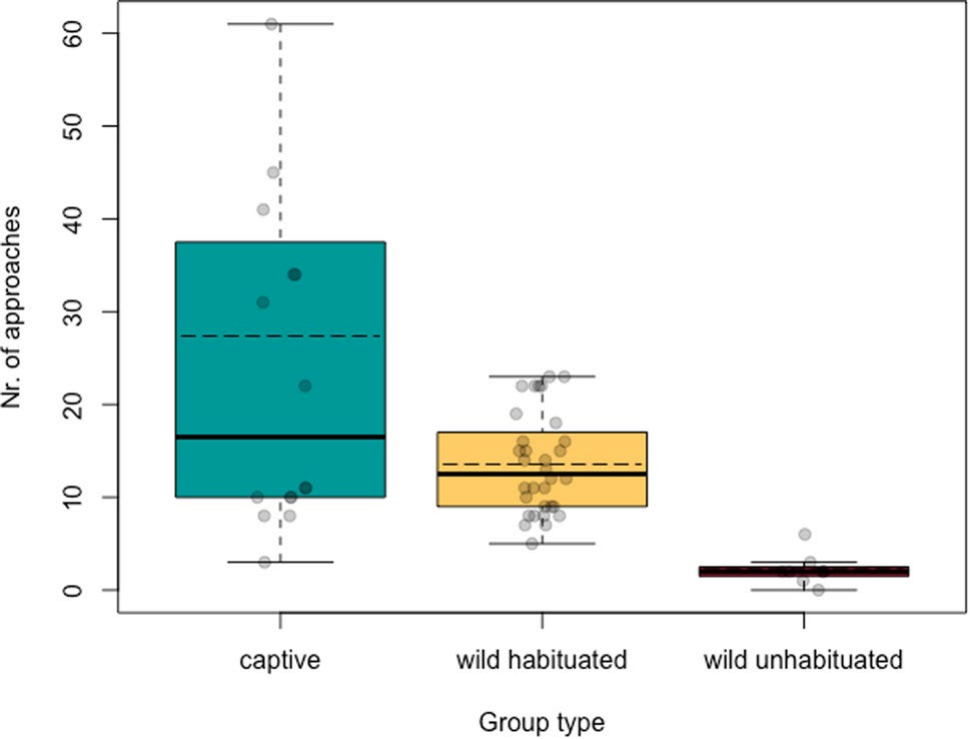
Boxplots of the number of approaches performed by each group type. Each point corresponds to a trial (*N*_captive_ = 16, *N*_wild habituated_ = 32, *N*_wild unhabituated_ = 8). Dashed lines correspond to the group means and solid lines correspond to the group medians

Model 1a was overall significantly different from its corresponding null model (likelihood ratio test: *X*^2^ = 70.94, df = 9, *p* < 0.001; *R*^2^ full model = 0.5; SI: Table 3S). Group type and stimuli type both had significant effects on monkeys’ approaches to the novel stimuli (group type: df = 2, *p* < 0.001; stimuli type: df = 7, *p* < 0.001). More specifically, we found that the three group types significantly differed among them (Fig. 1), with captive groups presenting the highest average number of close approaches to the novel objects and foods (captive–wild habituated: *p* < 0.001, Hedge’s *g* = 0.89; captive–wild unhabituated: *p* < 0.001, Hedge’s *g* = 1.16; wild habituated–wild unhabituated: *p* = 0.048, Hedge’s *g* = 2.23).

**Fig. 2:**
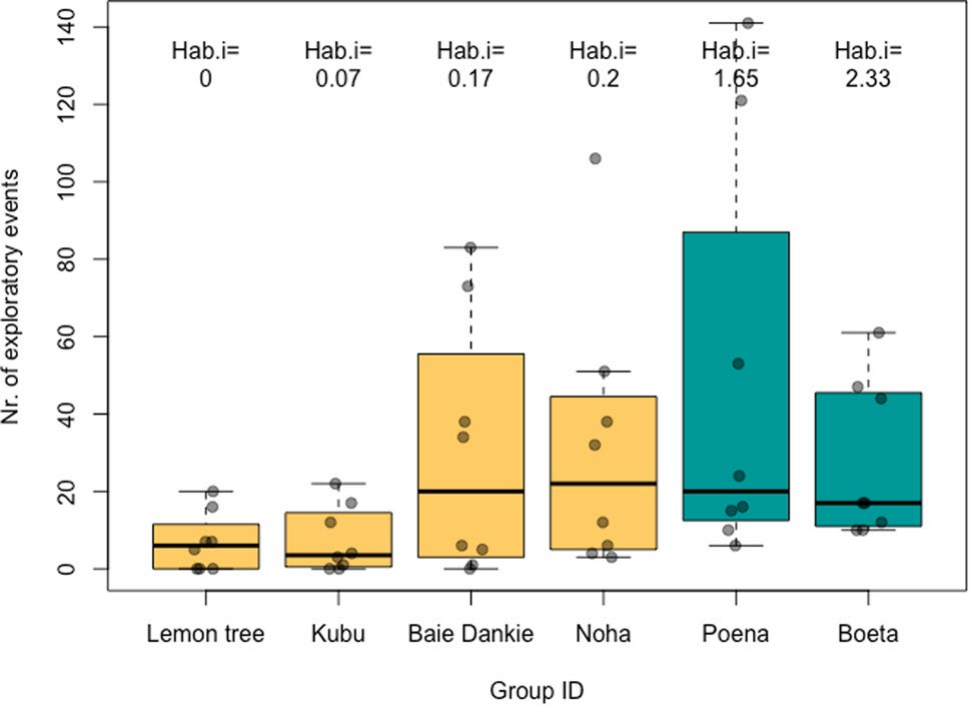
Boxplots of the number of exploratory events observed in each of the groups. Hab.i represents the habituation index calculated for each group. Green boxes correspond to the captive groups and yellow boxes correspond to wild-habituated groups

The visualization of the effects of stimuli type on the number of approaches by group (SI: Fig. 2S) suggested that the differences among stimuli indicated by the model were driven by the high number of approaches in the largest captive group (Poena). To determine if this was the case, we fitted Model 1a again, but removed the data from the Poena group. In this case, we found that although the full-null model comparison was significant (likelihood ratio test: *X*^2^ = 20.92, df = 9, *p* = 0.013; *R*^2^ full model = 0.41) and the significant effect of group type remained (*p* = 0.002), stimuli type did not have a significant effect on the number of approaches (*p* = 0.34).

Model 1b (SI: Table 3S) was overall significant both when the Poena group was included and excluded (with Poena: likelihood ratio test: *X*^2^ = 66.83, df = 9, *p* < 0.001; *R*^2^ full model = 0.5; without Poena: likelihood ratio test: *X*^2^ = 18.18, df = 9, *p* = 0.03; *R*^2^ full model = 0.41). In neither case did the habituation index (with Poena: *p* = 0.84, without Poena: *p* = 0.19) nor the group type (with Poena: *p* = 0.08, without Poena: *p* = 0.09) have significant effects on the number of close approaches observed in the different groups.

### Factors influencing exploration tendency

**Fig. 3:**
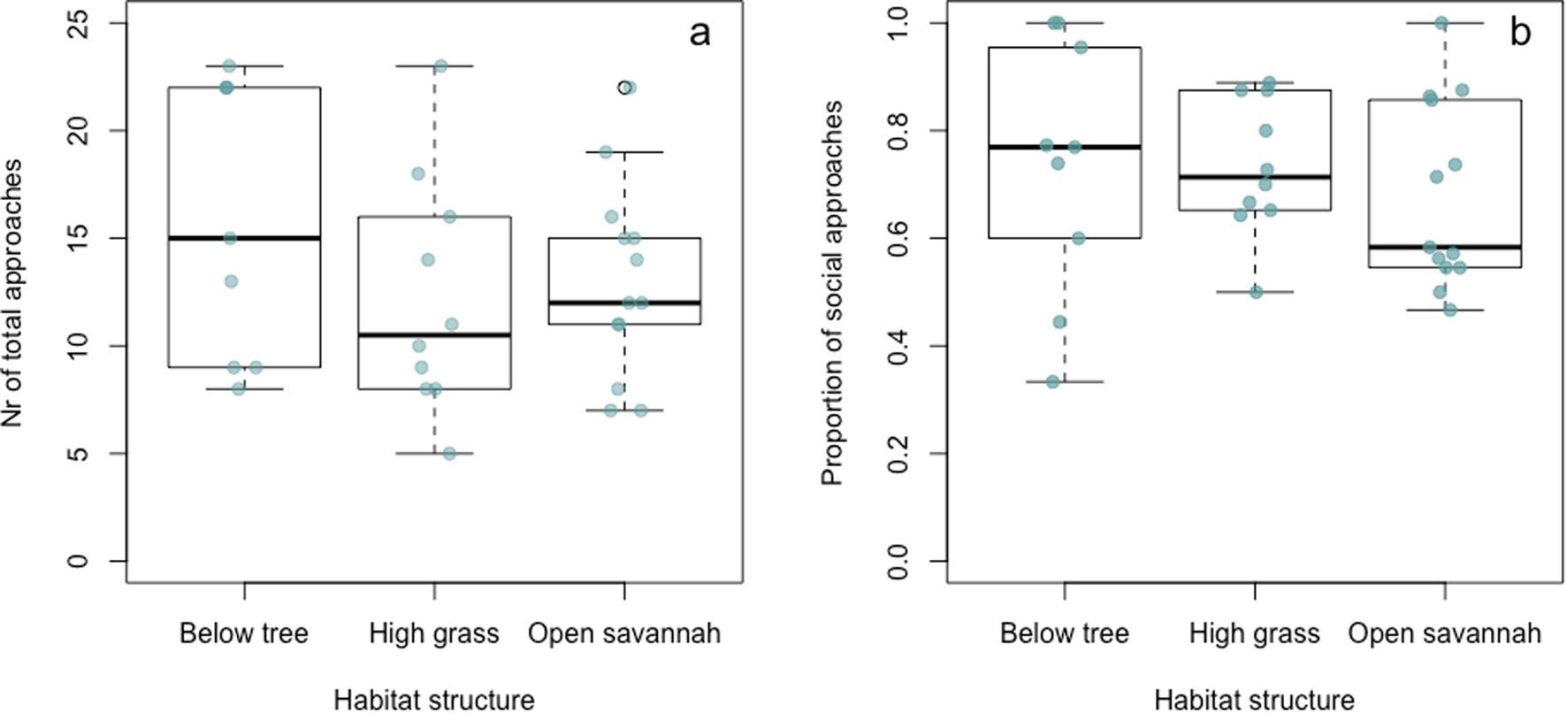
**a** Boxplots of the number of total close approaches observed in the different habitat structures and **b** the proportion of social approaches out of the total number of approaches (individual and social) observed in each of the experimental locations featuring different habitat structures. Each dot corresponds to a trial

Model 2 was overall significant according to the full-null model comparison (likelihood ratio test: *X*^2^ = 67.28, df = 9, *p* < 0.001; *R*^2^ full model = 0.68, SI: Table 5S). All test predictors had a significant effect on the response (habituation index: *p* < 0.001, Fig. 2; stimuli type: *p* = 0.001), although the significance of group type (i.e., difference in exploration events between captive and wild-habituated groups) was marginal (*p* = 0.047, Hedge’s *g* = 0.58). Visual assessment of the data suggested that the statistical differences in exploration tendency based on stimuli type were not driven by a particular group (SI: Fig. 3S). Differences in exploratory events based on stimuli type were investigated by changing the predictor's reference category (SI: Fig. 3S, Table 6S).

### Habitat structure and novelty approaches

Model 3 was overall significant according to the full-null model comparison (likelihood ratio test: *X*^2^ = 30.29, df = 10, *p* < 0.001; *R*^2^ full model = 0.68, SI: Table 7S). We found that the proportion of social approaches varied significantly across stimuli types (*p* < 0.001). However, the proportion of social approaches did not significantly differ based on habitat structure (*p* = 0.47, Fig. 3) or habituation index (*p* = 0.99).

## Discussion

### The effect of human habituation on curiosity

As opportunistic foragers, we would expect vervet monkeys to show relatively low neophobia to optimize their foraging niche (Greenberg and Mettke-Hofmann 2001; Greenberg 2003; Mettke-Hofmann 2014; Barrett et al. 2019). Our results, however, showed that within this species, neophobia levels were conditional on environment (captive and wild) and habituation level (Fig. 1). Wild individuals approached novel stimuli significantly less than captive conspecifics and, as predicted, within the wild sample, unhabituated monkeys approached novel items less than habituated individuals (Fig. 1). This contrasts with both the “*free time”* and *“excess energy”* hypotheses (Kummer and Goodall 1985; Laland and Reader 1999; Reader and Laland 2001; Amici et al. 2020), which would predict that both types of wild vervet groups (habituated and unhabituated) have similar approach frequencies, since they live in the same environment and therefore experience similar predation pressure, food abundance, and presumably are in need for similar amount of foraging and vigilance activities. Moreover, we exposed the wild unhabituated group to the novel stimuli longer than the habituated monkeys, due to the setup by the video camera traps. Thus, the need for wild individuals to attend to other activities during the experiments cannot account for the observed differences in the number of approaches between these group types. As such, our data do not support the “*free time”* or “*excess energy hypotheses”*. Rather, we propose *the habituation hypothesis* as a possible explanation of our findings, and discuss this more below.

Besides differences in the number of close approaches between monkeys from captive and wild habitats, the wild-habituated monkeys made an intermediate number of approaches, in-between their captive and wild unhabituated conspecifics (Fig. 1). The captive monkeys in our sample had never (or only at very early age) experienced any negative reinforcement when approaching anything unfamiliar as they spent all their life within a risk-free, food provisioned habitat and thereby probably have a positive perception of humans. This experience was reflected in the results of the habituation test, as almost all captive monkeys approached the man to the closest possible distance. Of course, we cannot account for the fact that the captive monkeys experienced a barrier between them and the unknown human as he was standing outside the enclosure mesh, however given that the more habituated-wild monkeys also approached to same distance suggest that habituation to humans and/or human artefacts reduces approach neophobia. In contrast to the captive monkeys, the wild monkeys at IVP are exposed to both negative and positive human interactions. Besides researchers (which are distinguished by their blue caps) who sporadically provide food through field experiments, they occasionally encounter poachers, hunters, and people living in villages just outside the reserve fence. Accordingly, the wild-habituated monkeys in our sample have become accustomed to humans and human artefacts but also experience the hazards of natural environments. It is possible that during the experiments, the wild-habituated monkeys perceived researcher presence as a safety indicator, or associated us with occasional feeding opportunities, which possibly raised their motivation to approach the novel stimuli compared to the unhabituated group. Yet, within the sample of habituated-wild monkeys, habituation index did not predict the number of approaches (SI: Table 4S and Fig. 2S), but groups with higher habituation indices had stronger exploration tendencies (Fig. 2). It is also worth emphasising that the majority of the habituated IVP monkeys avoid very close proximity even to familiar humans (Erica van de Waal, personal observation). These findings imply that a significant effect of habituation is the increased motivation to interact and manipulate novel stimuli, rather than just daring to come closer to humans or their artefacts, or expecting to obtain food from them. All together, these findings support our hypothesis that habituation to humans and/or their artefacts facilitates curiosity towards novelty in vervet monkeys.

Going beyond this, within the wild-habituated groups, we found lower explorative tendencies in Lemon Tree and Kubu compared to Baie Dankie and Noha. Indeed, the human-related experiences vary between the habituated groups. The home range of Lemon Tree is located furthest away from the IVP station; and in the previous years, both Lemon Tree and Kubu have encountered hunters/villagers more frequently than other groups. During the habituation process of the monkeys at IVP, Lemon Tree showed a delay in their habituation compared to the other habituated groups (Erica van de Waal, personal communication). Thus, it is plausible that the effect of human habituation on novelty responses is relative to the ratio of neutral-positive (researcher) encounters to neutral-negative (non-researchers, poachers, and hunters) encounters experienced by a group. Furthermore, both Lemon Tree and Kubu have participated in fewer field experiments, and thereby experienced less exposure to manufactured materials and food rewards. Moreover, we found that the groups with the higher habituation indices (Poena, Boata, Noha, and Baie Dankie) explored the plastic cars and rubber butterflies more than the two groups with lower habituation index (Kubu, Lemon Tree) (SI: Fig. 3S). Although these groups have more experiences with colourful items and anthropogenic materials, they also explored seashells more than the other groups, an item that was novel but represents a completely natural material (SI: Fig. 3S). These observations suggest that it was not the material per se that captured their interest but rather that habituation brings about a general change in their curiosity towards unfamiliar items, showing strong support for our habituation hypothesis.

### Stimuli type and curiosity

The different stimuli types that we presented to the monkeys did not influence the number of close approaches observed across groups, implying that since all items were new to the monkeys of all groups, each individual needed to approach first to judge whether or not to engage in further exploration. The categorization of man-made/processed versus more naturalistic stimuli did not have any general effect on responses (SI: Fig. 3S and Fig. 2S). Instead, the data suggest that items that emit a characteristic odour (fish, meatballs, cat toy mice, and boiled pasta) might be less explored on average than non-smelly items. Furthermore, both captive and wild vervet monkeys seemed reluctant to taste the strong-smelling food items fish and meatballs. Former experiments introducing novel foods have demonstrated that it indeed takes vervet monkeys multiple exposures to novel food before they accept it as a food source (Canteloup et al. 2020, 2021) and sociality plays a role in that monkeys are more likely to eat novel food after first observing a conspecific do so (Pooja et al. in prep). Thus, it is likely that monkeys perceive an unknown smell as repulsive and therefore explored such items less. One could argue that popcorn emits similar levels of odour as boiled pasta, yet popcorn was explored much more by the monkeys, especially by the two groups Baie Dankie and Noha (SI: Fig. 3S). These groups regularly participate in field experiments rewarded with soaked corn, and thus, it is possible that the monkeys of Baie Dankie and Noha associated the smell of popcorn with soaked corn, and thereby had a more positive association with the smell of popcorn compared to the other odours. Future experiments should investigate further the effect that odour cues have on novelty responses and exploration tendencies.

### Habitat structure and novelty responses

Compared to the wild-habituated groups, it is worth noting that the experiments with the unhabituated wild group always took place underneath a familiar, frequently used sleeping tree, where the monkeys are presumably relatively safe from aerial predators, and with no high grass to obscure potentially hidden snakes, yet this did not seem to increase their motivation to approach (Fif.1). Furthermore, even though open savannah exposes vervet monkeys to large birds and areas of tall grass can hide predatory snakes (Seyfarth et al. 1980), habitat structure had no influence on the motivation to approach novelty during our experiments (Fig. 3a). Previous findings suggest that vervet monkeys at IVP vocalize to recruit social partners, especially close to the river (Mercier et al. 2017), proposing that monkeys experience the river bank as a high-risk area. Thus, distance to the river could potentially be a more relevant variable to assess the influence of habitat on novelty responses. In our sample, habitat structure had no effect on whether or not a monkey approached alone or in a social context (Fig. 3b). This finding was somewhat unexpected, given that sociality has been reported to reduce the risk involved in approaching something new (Stöwe et al. 2006; Moretti et al. 2015), and watching a conspecific interact with novelty also increases exploration tendencies (Forss et al. 2017). Of course, social influences may also constrain an animal’s motivation to interact with novel stimuli due to monopolization or potential fear of aggression from conspecifics.

### Study limitations

Our study was limited by the inclusion of a single unhabituated group (Congo). Clearly, multiple groups of this category would be needed to verify the effect of habituation across wild monkeys. Moreover, the fact that the Lemon Tree group showed as equally low habituation index as the Kubu group despite the fact that monkeys in Lemon Tree have been regularly exposed to researchers for 3 years more than Kubu raises the question of to what extent within group dynamics potentially influence the monkey’s response to novelty. Kubu is a small group with a large proportion of juveniles and in many species, vervet monkeys included, juveniles seem to be more explorative than adults (Fairbanks and McGuire 1993; Bergman and Kitchen 2009; Thornton and Samson 2012; Debeffe et al. 2013). Considering within group dynamics, it will also help to evaluate what effects life-history and sociality have on curiosity. Thus, in the future, we intend to investigate these data at the individual level to clarify how potential within group variation may also contribute to the observed pattern between groups.

## Conclusion

One way to detect curiosity in animals is to introduce something novel into their familiar environment and measure their motivation to overcome potential neophobia and explore it. In doing so, we found evidence that curiosity in vervet monkeys is expressed through a combination of reduced neophobia (willingness to approach into close proximity) together with a variety of explorative behaviours like smelling, touching, and tasting something previously unknown (Table 2). Our findings, that captive and wild-habituated vervet monkeys responded more positively towards unfamiliar items than unhabituated conspecifics, despite the fact that all wild monkeys are exposed to similar risks in their natural habitat, support our conclusion that the main driver of curiosity in our sample was habituation level to humans and human-made artefacts, rather than risk constraints or time constraints of life in the wild. Consequently, our findings highlight the importance to account for the captivity effect and habituation levels when conducting cognitive research across settings.

## Supplementary Information

Below is the link to the electronic supplementary material.

Supplementary file1 (DOCX 5710 kb)

## Data Availability

All data for these analyses can be found in the Open Science Framework with the link: https://osf.io/2cahn/?view_only=e1d66702bc544363b5ced2ae51d97af5.

## Code availability

The code employed in these analyses can be found in the Open Science Framework with the link: https://osf.io/2cahn/?view_only=e1d66702bc544363b5ced2ae51d97af5.
