# Supplementary material for: Captivity and habituation to humans raise curiosity in vervet monkeys
Source: Anim Cogn. 2021 Dec 2;25(3):671–82. doi: 10.1007/s10071-021-01589-y (PMC9107434; doi:10.1007/s10071-021-01589-y)
Supplement: Supplementary file 1 — Supplementary file1 (DOCX 5710 kb) [file 10071_2021_1589_MOESM1_ESM.docx]

**Captivity and habituation to humans raise curiosity in vervet monkeys**

Sofia Ingrid Fredrika Forss^1,2,3^, Alba Motes-Rodrigo^4^, Pooja Dongre^1,2^, Tecla Mohr^1,2^ & Erica van de Waal^1,2^

*^1^Department of Ecology and Evolution, University of Lausanne, Lausanne, Switzerland*

*^2^Inkawu Vervet Project, Mawana Game Reserve, KwaZulu Natal, 3115, South Africa*

*^3^Department of Evolutionary Biology and Environmental Studies, University of Zurich, Zurich, Switzerland*

*^4^Department of Early Prehistory and Quaternary Ecology, Eberhard-Karls-Universität Tübingen, Tübingen, Germany*

**Supplementary material**


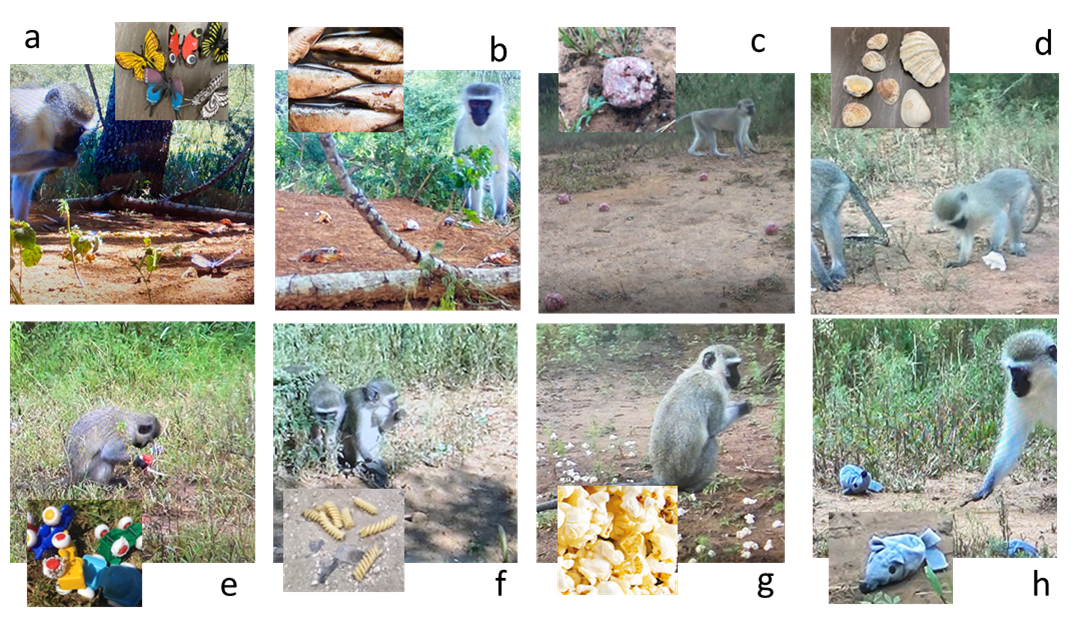


**Fig. 1S**: Pictures of the novel stimuli test battery: a) rubber butterflies b) canned fish c) minced meatballs d) seashells e) plastic cars f) boiled pasta g) popcorn and h) cat toy mice.

**Table 1S**: Ethogram explaining the definitions of the recorded exploration events.

| **Exploration event** | **Definition** |
| --- | --- |
| Smell | A smell event was recorded every single time a monkey put its nose in close contact (< 10 cm) to one of the presented stimuli. |
| Taste | Taste events were recorded every time a monkey licked an item of the presented stimuli, or every time a monkey put its lips onto a stimulus without taking the item into its mouth. |
| Touch | A touch event was defined by each time a monkey physically touched one of the stimuli presented with its hand or feet. These events were *not* recorded as any other event (smell, taste, chew/ bite, lift/ move). |
| Chew/ Bite | Chewing and biting was defined as one category of events, each time a monkey put a presented novel object, or parts of it, into its mouth and chewed on it or bit in it, without ingesting it. This excludes the events when monkeys were eating or tasting a novel food item. |
| Lift/ Move | Lifting or moving entails all events were a monkey removed a stimulus from the ground, lifted it up, turned it over as well as those events when a monkey carried an item with it to another location. |


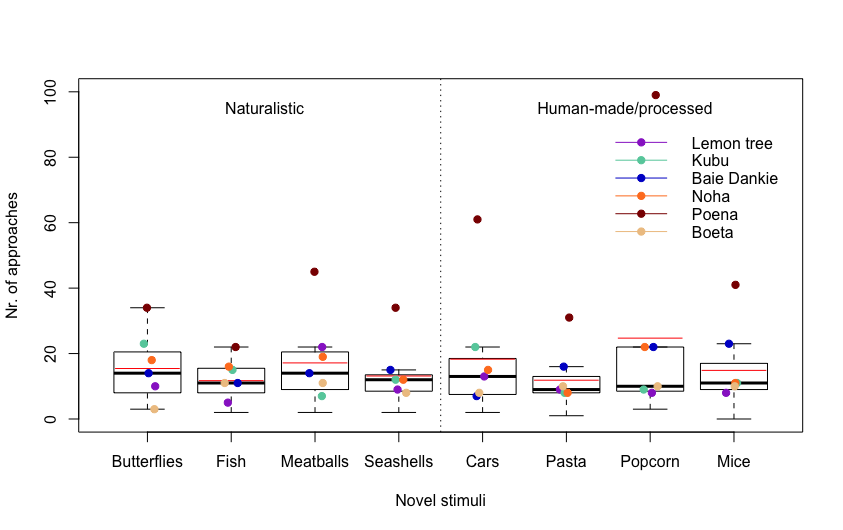


**Fig. 2S**: Boxplots of the number of approaches to each novel stimulus presented observed in each of the groups tested. Red horizontal lines represent group means and black horizontal lines represent group medians.


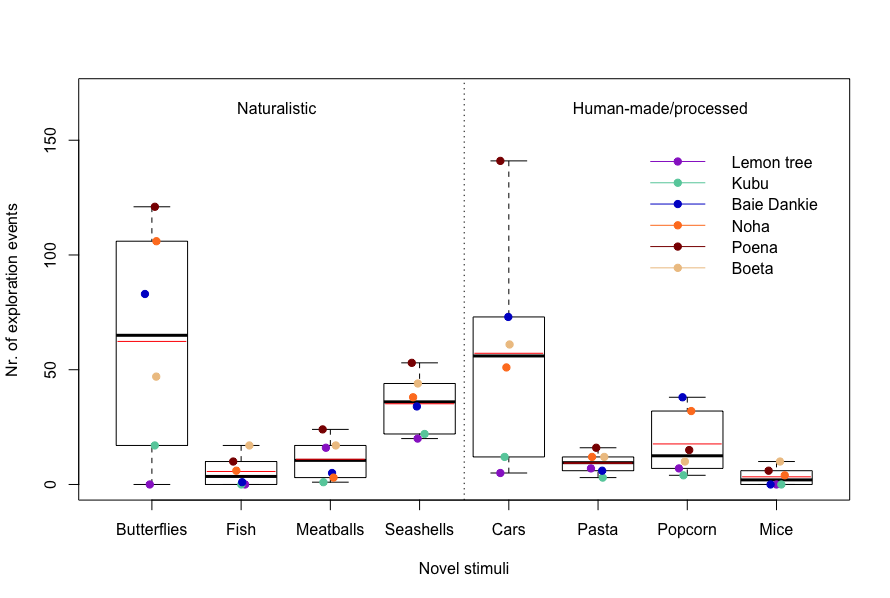


**Fig. 3S:** Boxplots of the number of exploratory events directed to the different novel stimuli presented to the different groups. Red horizontal lines represent group means and black lines represent group medians.


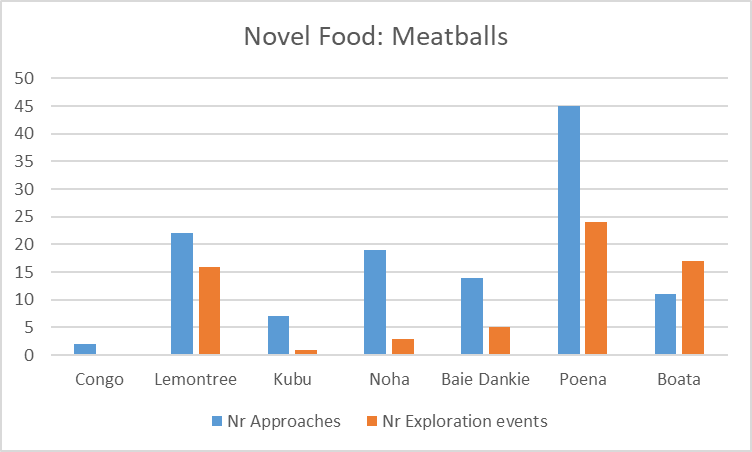
**a. b.**

**
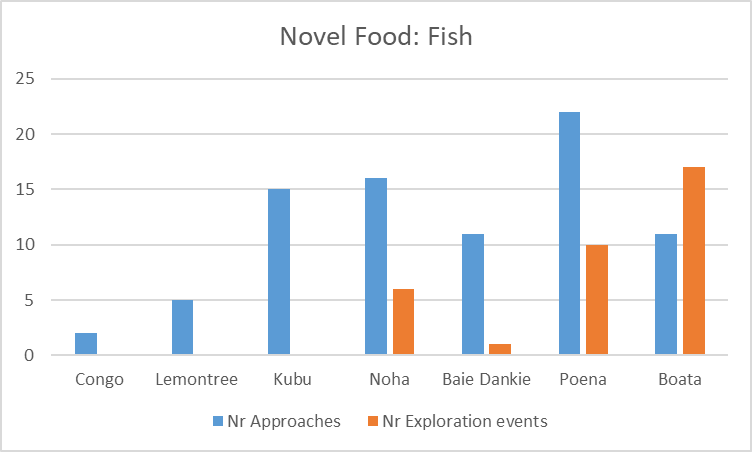
**

**
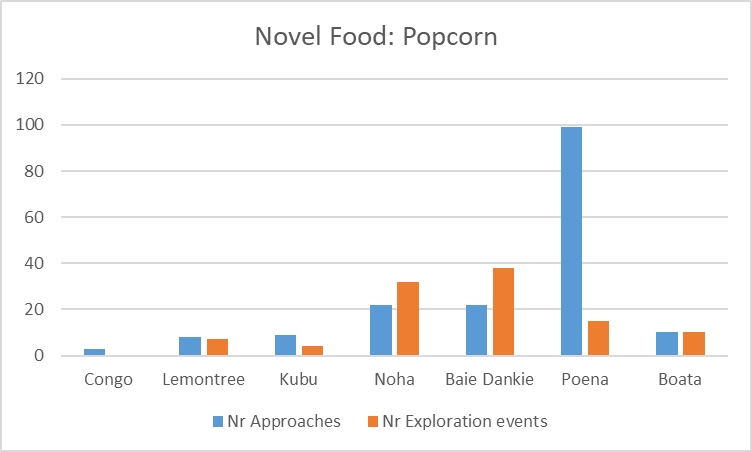

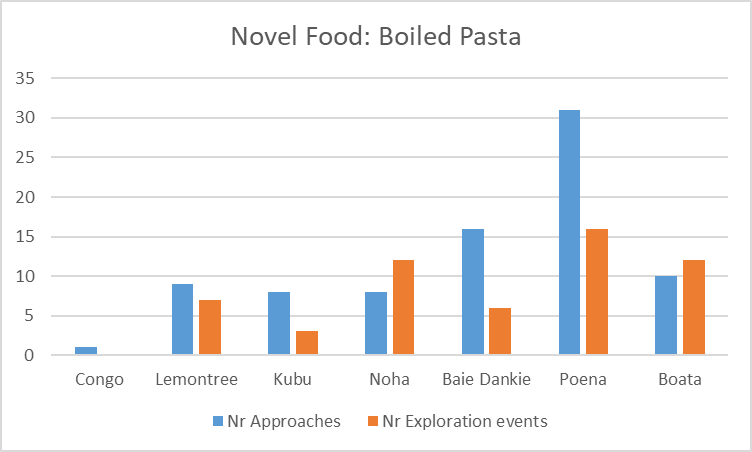
c. d.**

**
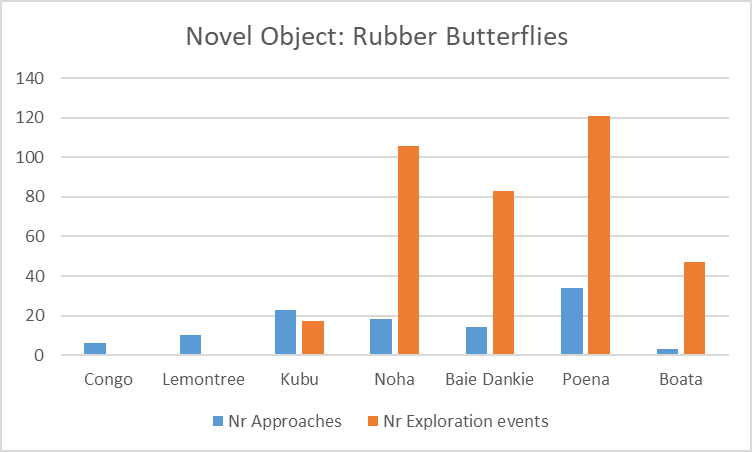

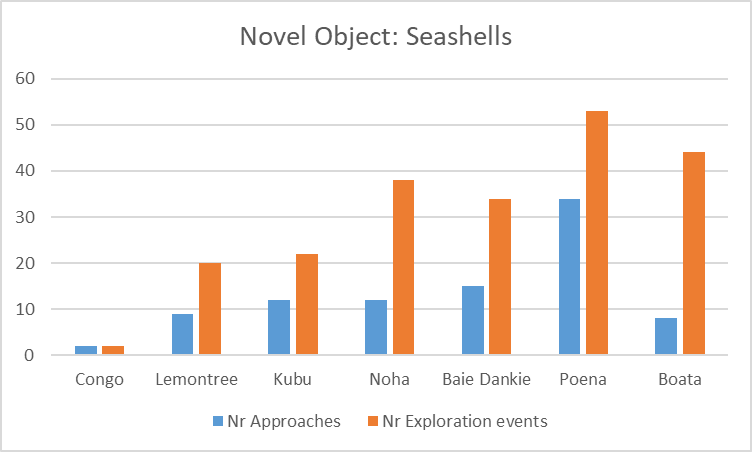
e. f.**

**g. h.**

**
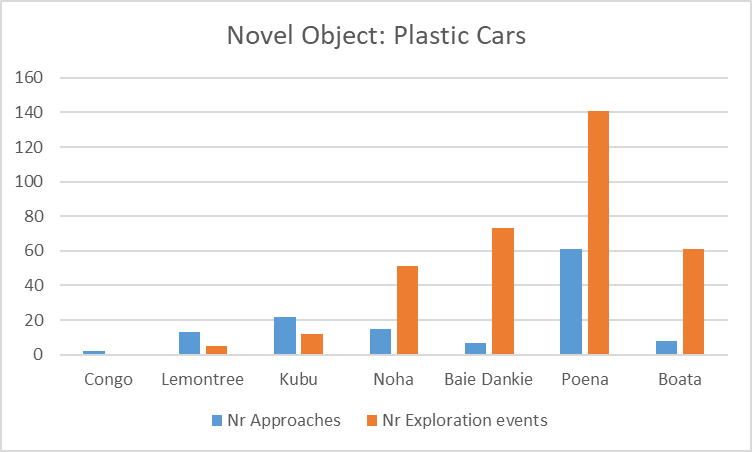

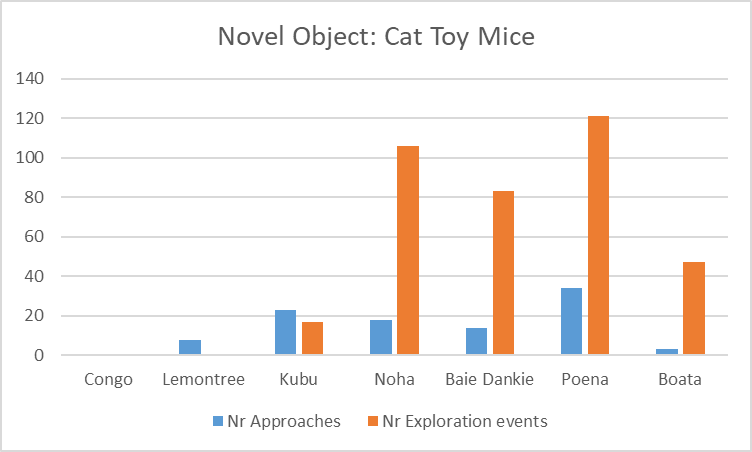
**

**Fig. 4S:** Raw data presenting the frequency of exploratory events and approaches per group and stimulus. Novel Food items: **a-d**, and Novel Objects: **e-h**.

**Model stability plots**

**Table 2S**: Overdispersion parameters estimated for each model.

| Model | Overdispersion parameter |
| --- | --- |
| 1a | 2.27 |
| 1b | 2.5 |
| 2 | 1.3 |
| 3 | 1.89 |


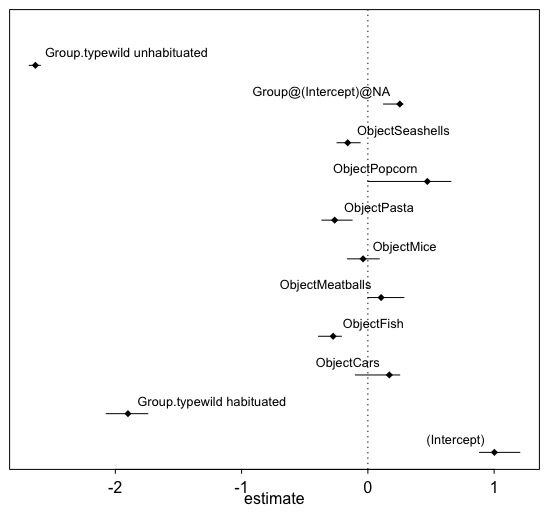

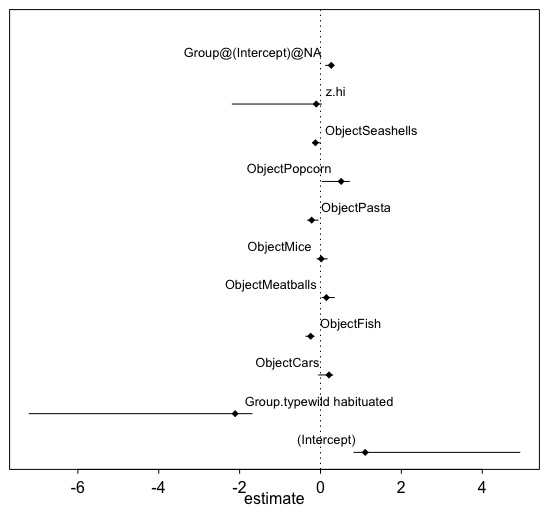
Model 1a Model 1b


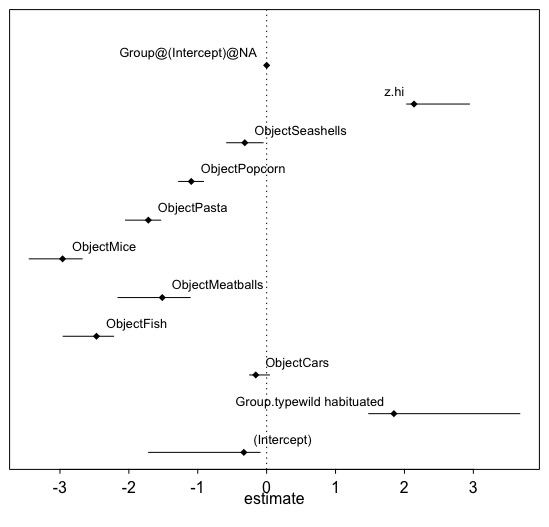

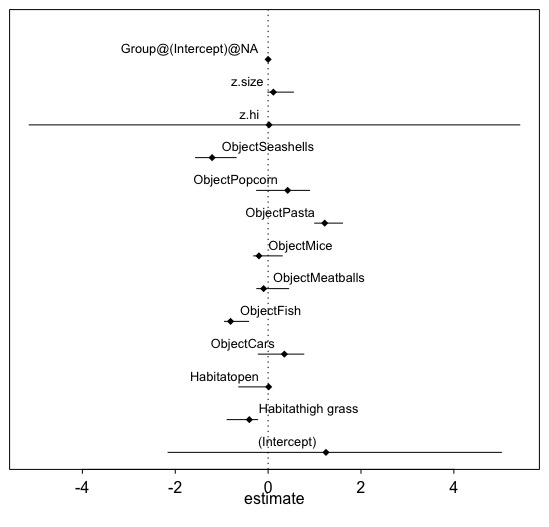
Model 2 Model 3

**Table 3S**: Fixed effects model estimates, standard errors, degrees of freedom and p values of Model 1a.

|  | Estimate | SE | df | p |
| --- | --- | --- | --- | --- |
| Intercept | 1.00 | 0.21 | a | a |
| Wild habituated^b^ | -1.90 | 0.23 | 2 | <0.001 |
| Wild unhabituated | -2.63 | 0.39 |  |  |
| Car^c^ | 0.17 | 0.13 | 7 | <0.001 |
| Fish | -0.28 | 0.15 |  |  |
| Meatball | 0.11 | 0.13 |  |  |
| Mice | -0.04 | 0.14 |  |  |
| Pasta | -0.26 | 0.15 |  |  |
| Popcorn | 0.47 | 0.12 |  |  |
| Seashells | -0.16 | 0.14 |  |  |

a: not shown for having limited interpretation

b: reference category was "captive"

c: reference category was "butterflies"

Random effects:

| Groups | Names | Variance | SD |
| --- | --- | --- | --- |
| Group ID (7) | Intercept | 0.06 | 0.25 |

Number of observations 56

**Table 4S**: Fixed effects model estimates, standard errors, degrees of freedom and p values of Model 1b.

|  | Estimate | SE | df | p |
| --- | --- | --- | --- | --- |
| Intercept | 1.11 | 0.72 | a | a |
| Wild habituated^b^ | -2.11 | 1.06 | 1 | 0.08 |
| Habituation index^c^ | -0.11 | 0.51 | 1 | 0.84 |
| Car^d^ | 0.21 | 0.13 | 7 | <0.001 |
| Fish | -0.24 | 0.15 |  |  |
| Meatball | 0.15 | 0.14 |  |  |
| Mice | 0.02 | 0.14 |  |  |
| Pasta | -0.22 | 0.15 |  |  |
| Popcorn | 0.51 | 0.13 |  |  |
| Seashells | -0.13 | 0.14 |  |  |

a: not shown for having limited interpretation

b: reference category was "captive"

c: z-transformed to mean of 0 and SD of 1. The mean and SD of the original variable were 0.73 and 0.99.

d: reference category was "butterflies"

Random effects:

| Groups | Names | Variance | SD |
| --- | --- | --- | --- |
| Group ID (6) | Intercept | 0.07 | 0.27 |

Number of observations 48

**Table 5S**: Fixed effects model estimates, standard errors, degrees of freedom and p values of Model 2.

|  | Estimate | SE | df | p |
| --- | --- | --- | --- | --- |
| Intercept | -0.33 | 0.64 | a | a |
| Wild habituated^b^ | 1.85 | 0.88 | 1 | 0.047 |
| Habituation index^c^ | 2.14 | 0.43 | 1 | <0.001 |
| Car^d^ | -0.16 | 0.36 | 7 | <0.001 |
| Fish | -2.47 | 0.41 |  |  |
| Meatball | -1.51 | 0.38 |  |  |
| Mice | -2.96 | 0.42 |  |  |
| Pasta | -1.72 | 0.39 |  |  |
| Popcorn | -1.09 | 0.37 |  |  |
| Seashells | -0.32 | 0.36 |  |  |

a: not shown for having limited interpretation

b: reference category was "captive"

c: z-transformed to mean of 0 and SD of 1. The mean and SD of the original variable were 0.73 and 0.99.

d: reference category was "butterflies"

Random effects

| Groups | Names | Variance | SD |
| --- | --- | --- | --- |
| Group ID (6) | Intercept | 0 | 0 |

Number of observations 48

**Table 6S**: Pairwise comparisons of the different novel stimuli presented to the different vervet groups. Each cell includes the p value of the difference between the two objects. Bold numbers indicate statistically significant differences.

|  | butterfly | cars | fish | meatball | mice | pasta | popcorn | seashell |
| --- | --- | --- | --- | --- | --- | --- | --- | --- |
| butterfly |  |  |  |  |  |  |  |  |
| cars | 0.67 |  |  |  |  |  |  |  |
| fish | **<0.001** | **<0.001** |  |  |  |  |  |  |
| meatball | **<0.001** | **<0.001** | **0.026** |  |  |  |  |  |
| mice | **<0.001** | **<0.001** | 0.29 | **0.001** |  |  |  |  |
| pasta | **<0.001** | **<0.001** | 0.08 | 0.62 | **0.006** |  |  |  |
| popcorn | **0.003** | **0.01** | **0.001** | 0.29 | **<0.001** | 0.12 |  |  |
| seashell | 0.38 | 0.66 | **<0.001** | **0.002** | **<0.001** | **<0.001** | **0.04** |  |

**Table 7S**: Fixed effects model estimates, standard errors, degrees of freedom and p values of Model 3.

|  | Estimate | SE | df | p |
| --- | --- | --- | --- | --- |
| Intercept | 1.25 | 1.50 | a | a |
| High grass^b^ | -0.40 | 0.40 | 2 | 0.47 |
| Open savannah | 0.01 | 0.37 |  |  |
| Habituation index^c^ | 0.02 | 2.19 | 1 | 0.99 |
| Group size | 0.11 | 0.19 | 1 | 0.54 |
| Car^d^ | 0.35 | 0.48 | 7 | <0.001 |
| Fish | -0.81 | 0.43 |  |  |
| Meatball | -0.10 | 0.45 |  |  |
| Mice | -0.20 | 0.43 |  |  |
| Pasta | 1.22 | 0.60 |  |  |
| Popcorn | 0.42 | 0.49 |  |  |
| Seashells | -1.21 | 0.46 |  |  |

a: not shown for having limited interpretation

b: reference category was "below tree"

c: z-transformed to mean of 0 and SD of 1. The mean and SD of the original variable were 0.11 and 0.09.

d: reference category was "butterflies"

Random effects:

| Groups | Names | Variance | SD |
| --- | --- | --- | --- |
| Group ID (4) | Intercept | 0 | 0 |

Number of observations 32
